# The Alternative Faces of Macrophage Generate Osteoclasts

**DOI:** 10.1155/2016/9089610

**Published:** 2016-02-08

**Authors:** N. Lampiasi, R. Russo, F. Zito

**Affiliations:** Institute of Biomedicine and Molecular Immunology “Alberto Monroy”, National Research Council, Via Ugo La Malfa 153, 90146 Palermo, Italy

## Abstract

The understanding of how osteoclasts are generated and whether they can be altered by inflammatory stimuli is a topic of particular interest for osteoclastogenesis. It is known that the monocyte/macrophage lineage gives rise to osteoclasts (OCs) by the action of macrophage colony stimulating factor (M-CSF) and receptor activator of nuclear factor-kB ligand (RANKL), which induce cell differentiation through their receptors, c-fms and RANK, respectively. The multinucleated giant cells (MGCs) generated by the engagement of RANK/RANKL are typical OCs. Nevertheless, very few studies have addressed the question of which subset of macrophages generates OCs. Indeed, two main subsets of macrophages are postulated, the inflammatory or classically activated type (M1) and the anti-inflammatory or alternatively activated type (M2). It has been proposed that macrophages can be polarized* in vitro* towards a predominantly M1 or M2 phenotype with the addition of granulocyte macrophage- (GM-) CSF or M-CSF, respectively. Various inflammatory stimuli known to induce macrophage polarization, such as LPS or TNF-*α*, can alter the type of MGC obtained from RANKL-induced differentiation. This review aims to highlight the role of immune-related stimuli and factors in inducing macrophages towards the osteoclastogenesis choice.

## 1. Introduction

**Figure 1:**
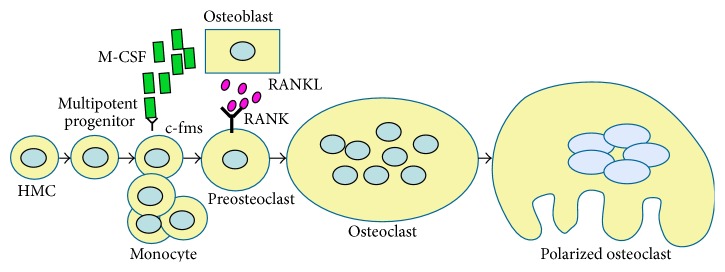
Osteoclastogenesis. Osteoclasts derived from monocyte/macrophage lineage after M-CSF and RANKL stimulation. Osteoclast precursors (preosteoclast) are M-CSF-dependent for proliferation and respond to RANKL through its receptor. Osteoblasts produce M-CSF and RANKL. Finally, preosteoclast fuse each other and differentiate into osteoclasts.

The vertebrate skeleton undergoes constant remodelling to remove old bone and maintain its structure throughout life. This entails a balance between bone synthesis by osteoblasts (OBs) and bone resorption by OCs. OBs are derived from mesenchymal stem cells (MSCs), while OCs arise from hematopoietic stem cells (HSCs) [1]. Briefly, OCs originate through a series of steps involving first the commitment of HSCs into the monocyte/macrophage lineage [2], then the proliferation of pre-OCs, their differentiation into OCs, and finally the cell polarization enabling resorptive activity [3]. Among the various colony-forming units (CFUs) giving rise to different cells of the myeloid lineages, only the CFU macrophages, in the presence of the M-CSF, undergo osteoclastic differentiation [4]. Ultimately, two osteoblast-derived cytokines control osteoclastogenesis, M-CSF, and RANKL [5, 6]. These cytokines engage their cognate receptors, c-fms and RANK, respectively, which are both present on pre-OCs [7] (Figure 1).

**Figure 2:**
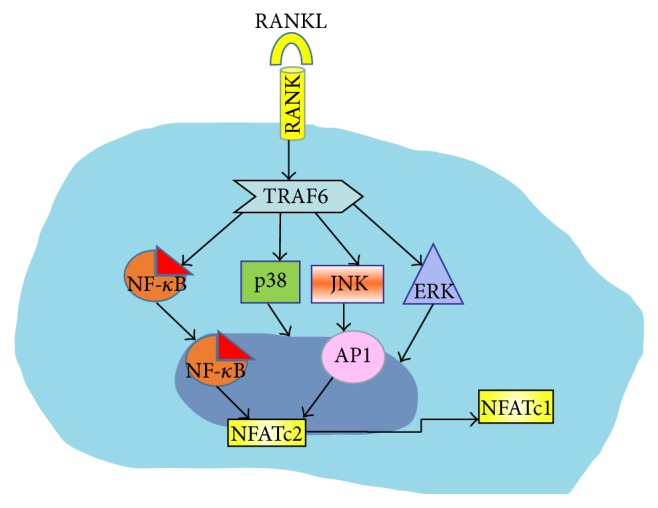
Preosteoclast. The interaction between RANK and RANKL induces the recruitment of TRAF6 leading to the activation of extracellular regulated kinase (ERK), p38 mitogen-activated protein kinase (p38), and c-jun N-terminal kinase (JNK). Therefore, transcription factors such as AP-1 and NF-*κ*B are activated and subsequently NF-*κ*B and NFATc2 are recruited to the promoter of* NFATc1*, which has been called the master regulator of osteoclastogenesis.

The interaction of RANKL with RANK induces the recruitment of tumor necrosis factor receptor-associated factors (TRAFs) to the cytoplasmic domain of RANK. This engagement leads to the activation of a signaling cascade with downstream targets including the extracellular regulated kinase (ERK), p38 mitogen-activated protein kinase (p38), c-jun N-terminal kinase (JNK), phosphatidylinositol-3 kinase (PI3K), and Akt and I*κ*B kinase [8, 9]. Consequently, some transcription factors, such as activator protein-1 (AP-1), nuclear factor-*κ*B (NF-*κ*B), and nuclear factor of activated T cells c1 (NFATc1), are activated. Finally, these transcription factors induce the expression of OC-specific genes, including tartrate-resistant acid phosphatase (TRAP) and cathepsin K [10, 11], and fusion-specific genes, including dendritic cell-specific transmembrane protein (DC-STAMP) and ATPase H1 transporting V0 subunit d isoform 2 (ATP6v0d2) (Figure 2).

Even though many extensive reviews have summarized data focusing on osteoclast origin, differentiation, and function, the precise identity of pre-OCs is not well known to date [12–19]. In this review, we will focus on recent progress in understanding the molecular basis underlying osteoclasts generation, including signaling pathways, transcription factors, and inflammatory stimuli coming from the microenvironment.

## 2. Macrophages Polarization

**Figure 3:**
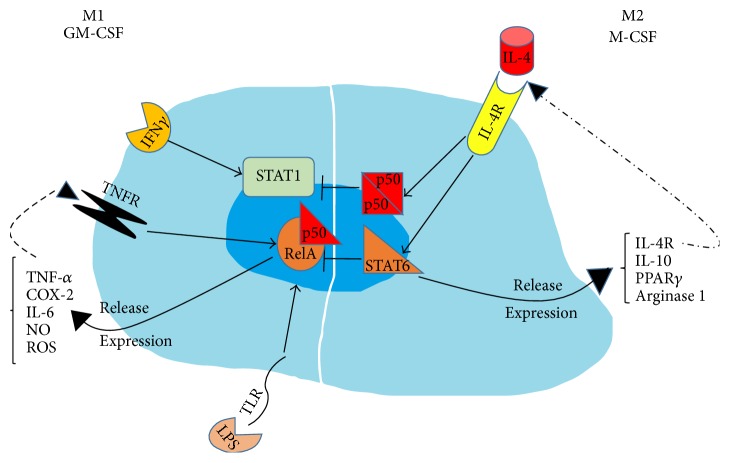
Macrophage polarization. Two cytokines GM-CSF and M-CSF contribute to the survival and proliferation of macrophages population in the steady-state conditions. TLR and IFN*γ* promote the expression of proinflammatory molecules such as TNF-*α*, IL-6, COX-2, NO, and ROS via STAT1 and NF-*κ*B (RelA/p50). In contrast, the activation of STAT6 and NF-*κ*B (p50/p50) by IL-4 promotes the expression of anti-inflammatory molecules such as IL-10, arginase 1, PPAR*γ*, IL-4R, and scavengers.

Macrophages are cells with great plasticity and versatility and appear very different from each other. Two cytokines, known as GM-CSF and M-CSF, are important for maintaining numbers and function of macrophages. They allow the steady-state condition in the macrophages development that can change depending on the presence of signals in the microenvironment. When macrophages are present in the tissues, they respond to signals with the acquisition of distinct functional phenotypes. Indeed, in response to ligands of toll-like receptors (TLRs) and IFN-*γ*, macrophages may undergo classical M1 activation, while they undergo an alternative M2 activation after stimulation by IL-4/IL-13. Actually, M1 and M2 represent two extremes in the spectrum of the macrophages phenotype. In fact, the analysis of surface antigens expression in human bone marrow shows a large number of different monocyte/macrophage phenotypes [20]. Similarly, data from the gene expression profiles reveal that murine macrophages from different organs are transcriptionally diverse, with minimal overlap [21]. The two-macrophage phenotype can be reversed, in some cases,* in vitro* and* in vivo* [22, 23]. Moreover, some inflammatory diseases are frequently associated with changes in macrophage activation, with the classically activated M1 cells implicated in initiating and sustaining inflammation and the M2 or M2-like cells associated with the resolution or dampening of inflammation [24]. In fact, M1 macrophages produce large amounts of proinflammatory cytokines, such as TNF-*α*, COX-2, and IL-6, can generate nitric oxide (NO) and reactive oxygen species (ROS), and express high levels of MHC molecules functioning as killer of pathogens and tumor cells. In contrast, M2 macrophages produce high quantity of IL-10, IL-4 receptor (IL-4R), and arginase 1, express scavenger receptors and molecules, and exhibit anti-inflammatory and tissue repair functions [25] (Figure 3).

Despite the large amount of data on macrophages diversity, the majority of transcription factors promoting alternative functional phenotypes, in response to different environmental inputs, are quite unknown. Among them, members of the signal transducer and activator of transcription (STAT) family together with interferon-regulatory factors (IRF) seem to play a key role in macrophage polarization. Indeed, the M1 macrophage phenotype is controlled by STAT1 and IRF5, whereas STAT6, IRF4, and peroxisome proliferator-activated receptor-*γ* (PPAR*γ*) regulate M2 macrophage polarization [25] (Figure 3). Another important transcription factor is PU.1, which determines macrophages identity through the regulation of their whole gene expression profile [26]. Furthermore, it has been demonstrated that PU.1 is involved in osteoclastogenesis, given that the development of macrophages and the OCs differentiation are arrested in PU.1-deficient mice [27].

## 3. Factors Associated with Macrophages Polarization

Macrophages ability to adapt to different stimuli from the microenvironment relies on their response to cytokines, cell-cell interaction, and pathological states. In general, the factors that contribute to macrophages polarization affect their ability to become OCs.

Under noninflammatory conditions, tissue-resident macrophages largely exhibit an M2 phenotype that promotes tissue homeostasis and repair. Interleukine-10 (IL-10), which is a potent anti-inflammatory cytokine produced mainly by M2 macrophages, inhibits the early stages of osteoclastogenesis, preventing the differentiation of osteoclast progenitors in pre-OCs. In particular, IL-10 may act directly by reducing the expression of NFATc1 and preventing its nuclear translocation [28] or indirectly through the reduction of RANKL and M-CSF expression [29].

Another suppressor of osteoclastogenesis is the IL-4, known to promote the M2 phenotype. It inhibits the RANKL-induced osteoclast differentiation through the STAT6-dependent inhibition of NF-*κ*B [30–33] but does not inhibit the distinctive ability of macrophages to fuse each other and form multinucleated giant cells (MGCs) other than OCs [34]. Similarly, GM-CSF inhibits RANK-mediated osteoclastogenesis, although it is not critical for macrophage development, since mice lacking GM-CSF do not have notable defects in tissue macrophages [35–37].

Under inflammatory conditions, for example, following an infection, M1 macrophage activation is induced by pathogen associated molecular patterns (PAMPs) such as lipopolysaccharide (LPS) in cooperation with interferon gamma (IFN*γ*) [38]. In this situation too, macrophages do not form OCs. Indeed, after the addition of highly purified LPS/IFN*γ*, bone marrow macrophages or RAW 264.7 macrophages (the last are considered as pre-OCs) fuse to form MGCs with specific immunological roles, while they neither express OC-specific enzymes nor show bone resorption [39]. Furthermore, the cell-cell fusion occurring after treatment with LPS/IFN*γ* of RANKL pretreated macrophages leads to the formation of multinucleated Langhans type giant cell (LGC) that do not show OCs characteristics [34]. Thus, RANKL treatment of RAW 264.7 macrophages does not commit them to become OCs when they are subjected to LPS/IFN*γ* or IL-4 treatment [34], but it generates two other forms of M1-derived MGCs showing specific immunological roles.

When inflammation is a consequence of infection or tissues injuries, TLRs are activated. The TLRs engagement leads to the NF-*κ*B activation and production of inflammatory mediators by M1 macrophages [40], but at the same time, TLRs promote the resolution of inflammation through the IKK/NF-*κ*B signaling pathway associated with M2 polarization [41].

The plasticity of macrophages to switch from M1 to M2 phenotype and* vice versa* and their potential differentiation in OCs might depend on the activation of different NF-*κ*B subunits and/or on its temporal activation. NF-*κ*B is considered the master regulator of inflammation, but at the same time it has gained importance in osteoimmunology. In fact, an essential role for NF-*κ*B in osteoclastogenesis was discovered when double-knockout mice for NF-*κ*B p50 and p52 were generated and found to have severe osteopetrosis because of the lacking of OCs [42]. The p50 NF-*κ*B homodimers contribute to M2 polarization* in vitro* and* in vivo* [43]. The RelA/p50 NF-*κ*B pathway, known as the “canonical” one, is typically activated through engagement of receptors like RANK, TNFR, and IL-1R and depends on IKK*β* and IKK*γ* activities. In contrast, the RelB/p52 NF-*κ*B “noncanonical” pathway is activated only by RANK and requires IKK*α* [44]. A unique and novel cross talk has been demonstrated between the canonical and noncanonical NF-*κ*B pathway, which appears sufficient to induce osteoclast formation* in vitro* and bone loss* in vivo* [45].

Additionally, the two NF-*κ*B subunits RelA [46] and RelB [47] have been identified as regulators of osteoclast survival and differentiation, respectively. In fact, one of the earliest responses of pre-OCs to RANKL (1-2 hours after its addition) is the recruitment of RelA/p50 and NFATc2 by the promoter of* NFATc1*, which has been considered the master regulator of osteoclastogenesis. In turn, NFATc1 transiently autoamplifies its own expression [48], accompanied by the downregulation of constitutively active repressors of RANK signaling [49], thus allowing osteoclastogenesis to proceed (Figure 2).

## 4. OsteoMacs: The Bone Macrophages

In physiological conditions, the bone contains resident macrophages denominated “OsteoMacs” that contribute to homeostasis and repair of the tissue and increase their number in the presence of active bone anabolism [14, 50, 51]. These tissue-resident macrophages derive from Ly6c “resident” monocytes that pass from the circulation to tissues under homeostatic conditions. In mice, OsteoMacs express numerous myeloid lineage markers such as F4/80, CD115, Mac3, and CD68 but express very low if any osteoclast and inflammatory macrophage markers [50, 52]. In another study, two types of resident macrophages were found in the bone. The population of TRAP^+^F4/80^−^ cells expresses TRAP as a marker for OC activity and consequently promotes bone resorption, while a second population of cells, named F4/80^+^CD169^+^TRAP^−^, lacks OCs characteristics and promotes erythropoiesis [53]. Recently, a new population of pre-OCs has been identified, which shows a mix of M1- and M2-like phenotypes together with the ability to suppress T cells using NO-dependent mechanism [54]. These pre-OCs, phenotypically CD11b^−^/^lo^Ly6c^hi^, are a different population with respect to the previously identified, since they retain some plasticity to differentiate into macrophages or dendritic cells (DCs) in the appropriate cytokine environment.

In case of inflammation due, for example, to bone fracture, macrophages are recruited in both humans [55] and animals [56]. These macrophages arise from a distinct population of blood monocytes, which rapidly infiltrate tissues compromised by the injury [57] and produce inflammatory cytokines generally associated with M1 phenotype. It has been reported that recruited inflammatory macrophages are derived from Mac3^+^F4/80^−^ monocytes and can differentiate in OCs under inflammatory conditions, although they are not considered precursors of OCs under homeostatic conditions. Therefore, the inflammatory microenvironment affects OCs differentiation via the actions of multiple cytokines and several studies suggest that pre-OCs can be also altered by inflammation. Nevertheless, it cannot be assumed that recruited inflammatory macrophages will contribute to bone repair.

## 5. Lessons from Inflammatory Pathological Conditions

Inflammation is a complex set of events that includes in itself both the origin and the end of the process. This implies that proinflammatory cytokines and transcription factors can initiate the reaction and at the same time promote its switching off. In the microenvironment of the bone tissue, in inflammatory and/or pathological conditions, there exist signals able to promote the phenotype M1 and at the same time to induce the switch towards the M2 phenotype. The emerging phenotype greatly depends on the time window during which the stimulating signals act as well as on the target cell type (i.e., macrophages, pre-OCs, or other).

A great deal of knowledge about the origin of OCs comes from the studies on diseases and inflammatory diseases such as rheumatoid arthritis (RA) and periodontitis.

**Figure 4:**
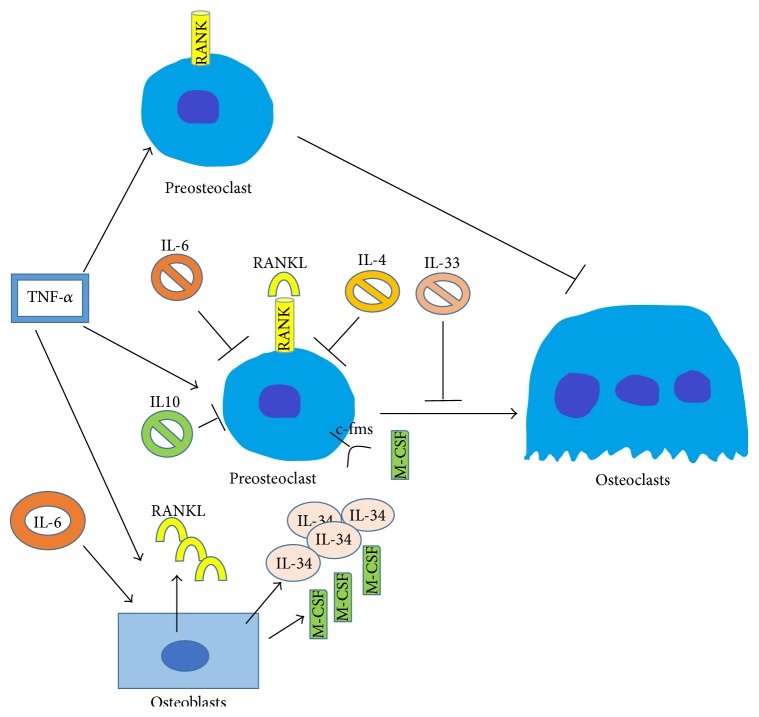
Effect of cytokines on the generation of osteoclasts. TNF-*α* promotes osteoclastogenesis directly, increasing the number of osteoclasts, and indirectly, increasing RANKL production by osteoblasts. The exposition of preosteoclast to TNF-*α* before RANKL inhibits osteoclastogenesis. IL-6 promotes osteoclast differentiation in the presence of osteoblasts and can synergize with TNF-*α* to induce osteoclastogenesis. On the contrary, it directly inhibits the differentiation of preosteoclast promoting the production of M1-like macrophage markers. IL-34 is produced by osteoblasts and recognizes the receptor for M-CSF (c-fms), thus promoting osteoclasts differentiation. IL-33 inhibits osteoclastogenesis addressing the preosteoclast versus M2-like macrophage differentiation. IL-10 is a potent anti-inflammatory cytokine produced mainly by M2 macrophages, which prevent the differentiation of osteoclast progenitors in preosteoclast. IL-4 promotes the M2 phenotype and inhibits the RANKL-induced osteoclast differentiation.

An increased number of circulating pre-OCs can be detected in psoriatic arthritis and RA patients in which the TNF-*α* levels are very high [58, 59]. TNF-*α*, IFN-*γ*, and other proinflammatory cytokines have been shown to promote osteoclastogenesis both directly, by increasing the number of pre-OC and/or their differentiation, and indirectly, via OBs and other stromal cells, which increase RANKL production [60, 61]. TNF-*α* generally stimulates M1 differentiation [62] and can promote the switch of M-CSF-primed M2 into M1 phenotype. It has been reported that TNF-*α* and LPS induced the formation of OCs from pre-OCs, suggesting that these factors can induce OCs fusion rather than their differentiation [63]. Otherwise, the effect of TNF-*α* is observed in the late phase of OC differentiation characterized by NFATc1 autoamplification [48]. Interestingly, the exposition of pre-OCs to TNF-*α* before RANKL results in the inhibition of osteoclastogenesis, possibly because TNF-*α*-stimulated pre-OCs are disposed to commit themselves towards activated macrophages [64] (Figure 4). However, osteoclastogenesis is promoted when TNF-*α* is added after RANKL or when pre-OCs are costimulated by TNF-*α* and RANKL [48]. Other inflammatory cytokines can affect OC development in pathological conditions. In* in vitro* experiments, the pleiotropic cytokine IL-6 indirectly promotes OCs maturation and activation depending on the presence of OBs that, ultimately, produce RANKL [65] (Figure 4). Transgenic mice overexpressing IL-6 showed osteopenia, unbalanced bone formation, and resorption probably due to an increased number of OCs and decreased number of OBs, suggesting a role as a direct inducer of osteoclastic formation [66]. IL-6 is present in the serum of RA patients and has an important role in increasing osteoclastic activity and subsequent bone resorption [67]. Indeed, IL-6 synergizes with TNF-*α* to induce osteoclastogenesis and bone resorption [68]. However, it has been reported that IL-6 targets pre-OCs and inhibits RANKL-induced osteoclastogenesis, increasing the expression of macrophage-specific markers, such as CD11b and Emr1, suggesting that IL-6 induces a macrophage phenotype instead of an osteoclastic one [69] (Figure 4).

Very recently, the novel cytokine IL-34 was shown to stimulate the viability of monocytes and CFU-M from BMCs. The receptor for IL-34 corresponds to the already known receptor for M-CSF [70]. IL-34, together with RANKL, induces the formation of OCs from splenocytes as well as from BMCs in mouse, while in humans it promotes the OC differentiation from peripheral blood mononucleated cells. Therefore, this cytokine can replace M-CSF for the differentiation of OCs both in mouse and in humans. IL-34 is produced by OBs [71], suggesting that these cells not only are devoted to the bone formation but also play an important regulatory role in bone homeostasis by producing such cytokines needed to coordinate the differentiation process of bone resorbing OCs [18].

IL-33 is a cytokine belonging to the recently discovered IL-1 family [72], which is constitutively expressed in various tissues and released under inflammatory conditions. Among others, IL-33 has been described to amplify polarization of M2 macrophages [73] and to skew the induction of pre-OCs toward M2 phenotype thus inhibiting their differentiation in OCs [74]. Moreover, IL-33 inhibits TNF-*α*-mediated bone destruction* in vivo* and directly inhibits early RANKL-induced osteoclastogenesis* in vitro* [74]. Recently, it has been reported that IL-33 acts by interfering with RANKL-mediated nuclear translocation of NFATc1 [75].

Interestingly, in inflammatory conditions, it has been shown that RANKL generates ROS in both OCs and their precursors to promote their differentiation and bone resorption [76, 77]. Indeed, it is known that small nontoxic amounts of ROS may play a role as second messenger in various signaling pathways, and thus, it can behave as an intercellular signal mediator for optimal differentiation and function of OCs. Very recently, it has been demonstrated that a new protein, named the negative regulator of ROS (NRROS), inhibits ROS production in phagocytes during inflammatory response [78]. The overexpression of NRROS in M-CSF-primed BMM attenuates the expression of all the osteoclastic genes induced by RANKL; in particular NRROS inhibits NF-*κ*B activation by blocking the degradation of I-*κ*B proteins [79].

An interesting molecule involved in the regulation of inflammatory events, such as obesity and type 2 diabetes, is GPR120, a G protein-coupled receptor engaged by unsaturated long-chain fatty acids [80]. It is involved in the differentiation of macrophages, chondrocytes [81, 82], and OBs [83, 84]. Very recently, a study demonstrated that GPR120 has a role in bone metabolism and reverses the process of bone loss through OBs activity [85]. Another study reported the GPR120 expression in OCs and in a lesser grade in their progenitors (BMMs), suggesting its negative role in osteoclast differentiation and bone resorbing activity, probably through the suppression of NFATc1 and inhibition of IkB*α* [86].

In conclusion, such a dynamic situation is actually fixed by a static photograph that heavily limits the definitive identification of the precursor of OCs.

## 6. Concluding Remarks

The treatment of macrophages with cytokines initiates a signal cascade that results in differential modulation of different genes. The differentiation of macrophages induced by subset of cytokines does not seem stable and irreversible, but rather it is transient and dependent on the microenvironment, as recently reported by studies that found a common progenitor for macrophage, dendritic, and osteoclast cells in human and mouse [87, 88].

In the bone, depending on the factors present in the microenvironment and on the cell-cell interactions, the macrophages are polarized toward M1 or M2 phenotype. It should be taken into account that* in vivo* macrophages are simultaneously exposed to a plethora of agents, which are capable of affecting their functional and phenotypic characteristics. Thus, it remains to be determined to what extent distinct subpopulations exist* in vivo*. Recent attempts have been made to reclassify macrophage subpopulations taking into account the many stimuli and the increasingly complex combination of markers present on their membrane [89]. However, different macrophages cannot easily be binned into defined groups.

The evidence given so far leads to the conclusion that the resident macrophages in the bone tissue with a M2 phenotype (OsteoMacs) can change their phenotype into M1-like under conditions of stress or bone fracture. However, the relationship between M1 macrophages phenotype and OCs is not always true. In addition, the recent literature regarding the formation and destruction of bone tissue shows that OsteoMacs are conditioned by the presence of their MSC precursors, which promotes differentiation and survival of OBs through oncostatin M. OBs, in turn, are among the most important regulators of bone tissue, as they also influence the differentiation and the survival of OCs. Despite the large number of data published on the subject, it is difficult to draw firm conclusions, because the* in vivo* situation can be very different from that studied on* in vitro* models. Furthermore, the situation under physiological conditions is certainly very different from that under inflammatory conditions, thus requiring additional studies to clarify the nature of OC precursors.
